# Early Life Microbial Exposure and Immunity Training Effects on Asthma Development and Progression

**DOI:** 10.3389/fmed.2021.662262

**Published:** 2021-06-16

**Authors:** Andressa Daronco Cereta, Vinícius Rosa Oliveira, Ivan Peres Costa, Letícia Lopes Guimarães, João Pedro Ribeiro Afonso, Adriano Luís Fonseca, Alan Robson Trigueiro de Sousa, Guilherme Augusto Moreira Silva, Diego A. C. P. G. Mello, Luis Vicente Franco de Oliveira, Renata Kelly da Palma

**Affiliations:** ^1^School of Veterinary Medicine and Animal Sciences, University of São Paulo, São Paulo, Brazil; ^2^Department of Physical Therapy, EUSES University School, University of Barcelona-University of Girona (UB-UdG), Barcelona, Spain; ^3^Research Group on Methodology, Methods, Models and Outcomes of Health and Social Sciences (M_3_O), University of VIC-Central University of Catalonia, Vic, Spain; ^4^Master's and Doctoral Programs in Rehabilitation Sciences, Nove de Julho University, São Paulo, Brazil; ^5^Human Movement and Rehabilitation, Post Graduation Program Medical School, University Center of Anápolis-UniEVANGELICA, Anápolis, Brazil; ^6^Institute for Bioengineering of Catalonia, Barcelona, Spain

**Keywords:** asthma, lung microbiome, dysbiosis, early life immunity, prevention strategies

## Abstract

Asthma is the most common inflammatory disease affecting the lungs, which can be caused by intrauterine or postnatal insults depending on the exposure to environmental factors. During early life, the exposure to different risk factors can influence the microbiome leading to undesired changes to the immune system. The modulations of the immunity, caused by dysbiosis during development, can increase the susceptibility to allergic diseases. On the other hand, immune training approaches during pregnancy can prevent allergic inflammatory diseases of the airways. In this review, we focus on evidence of risk factors in early life that can alter the development of lung immunity associated with dysbiosis, that leads to asthma and affect childhood and adult life. Furthermore, we discuss new ideas for potential prevention strategies that can be applied during pregnancy and postnatal period.

## Introduction

Asthma is the most common heterogeneous inflammatory lung disease appearing generally in childhood. Adults are also affected, and more than 339 million people of all ages are living with asthma worldwide. Over 80% of asthma-related deaths occur in low-and lower-middle income countries (1). The pathophysiology of asthma is complex including phenotypes (visible properties) and Endotypes (mechanisms). Regarding phenotypes, the most common are allergic, in early onset, mild, or moderate-to-severe remodeled asthma or non-allergic with late-onset eosinophilic asthma or non-eosinophilic asthma (2). In addition, several factors such environmental, genetic polymorphisms, epigenetic regulations, aberrant immune maturation during pregnancy, and other factors in early life can contribute to the development of asthma. Regarding these factors we can find respiratory infections (mainly the viral ones), the exposure to airborne environment agents (tobacco smoke, pollutants), and most recently comes to light the important role in microbiome imbalance (3).

In this sense, there is not a unique cause or major determinant risk factor that contributes to the development of asthma. Apparently, the combination of several factors in early life and inflammatory response due to it in a period of rapid growth and development of the lung causes structural and immune impairments that leading to asthma (4). Therefore, the key for developing prevention and strategies treatment in asthma is trying to understand the early-life exposures. In this review, we focus on evidence of risk factors in early life that can alter the development of lung immunity associated with dysbiosis, that leads to asthma and affect childhood and adult life.

## Events in Early Life and Dysbiosis in Asthma

**Figure 1:**
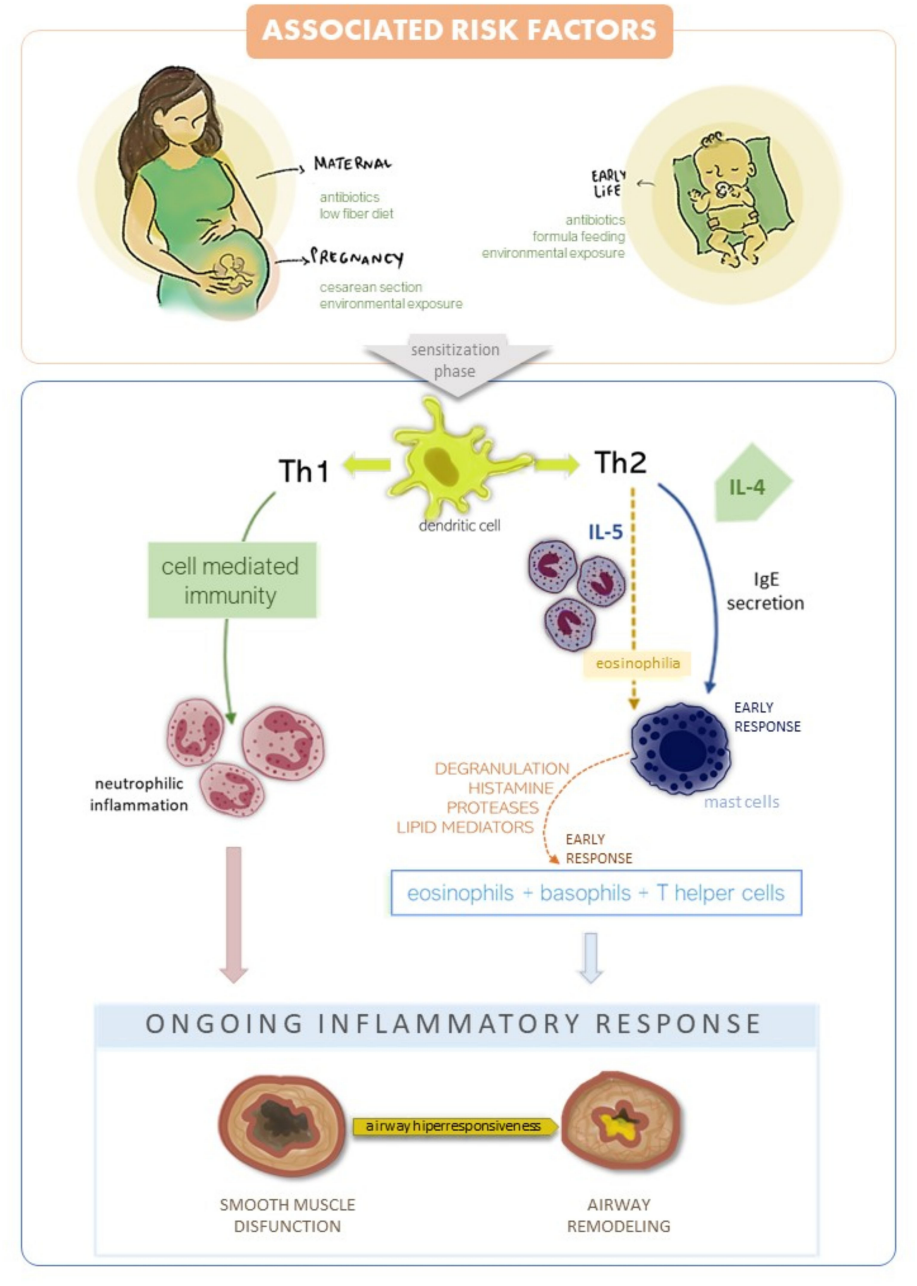
Associated risk factors and asthma development. There are two inflammatory responses in asthma: Th1 and Th2 mediated. Th2 is the most common and leads to an early response by IgE production, presence of eosinophilia and mast cell recruitment. Late response in Th2 mediated asthma recruits' eosinophils, basophils and T helper cells to the mucosa leads to inflammatory response. Th1 mediate cell immunity in asthma, with neutrophilic inflammation affecting the epithelial layer. Both Th1 and Th2 cause airway hyper responsiveness, smooth muscle disfunction and airway remodeling.

As above mentioned, asthma is developed due to several risk factors and can be linked with prenatal or early life events, causing it to appear specially in childhood. During early life, asthma can be associated with factors (Figure 1) such as delivery by cesarean section, antibiotics usage during the neonatal period, maternal low fiber diet, formula feeding, pollution and the variety of microbes due to environmental exposure (5). Therefore, perturbations on microbial composition (dysbiosis) can consequently alter immune development in mucosal tissues and lead to an increased susceptibility to asthma. Alterations in the microbiome in asthma are due to an association between changes in diversity and composition of lung microbiota along with modifications of functional genes (6). Besides that of the lung, nasal and bronchial microbiomes, asthmatic children also have alterations in the gut microbiome (7). Instances of crosstalk between gut and lung, called the “gut-lung” axis, have been demonstrated. For example, several studies have demonstrated that gut microbiome modulate Tregs in immune function by producing local and systemic mediators which impact on asthma development mediated by gut-lung axis (5, 8, 9).

In the recent years there were many advances in gene sequencing technology, expanding the knowledge on lung and gut microbiome, and on the significant role of the interactions between these two niches in the development and incidence of chronic airway disease. The airways are composed by a resident microbiota that develops after birth and interacts with different body sites, such as the gut, and its composition changes in health and disease (10).

Previous studies have suggested a strong correlation between mode of delivery and asthma incidence (11, 12). At the time of birth, maternal bacterial population is transferred to the baby. Stokholm et al. (13) demonstrated that vaginal delivery was associated with neonatal colonization of the intestinal tract by *Escherichia coli* at age 1 week while colonization by *Citrobacter freundii, Clostridium species, Enterobacter cloacae, Enterococcus faecalis, Klebsiella oxytoca, Klebsiella pneumoniae*, and *Staphylococcus aureus* were associated with cesarean section. However, at age 1 year this gut microbial perturbations were not apparent. Therefore, the same group conducted a cohort prospectively study with 700 children to investigate a risk of developing asthma in the first 6 years of life (14). Children who retained a cesarean gut microbial profile at age 1 year were more susceptible to developing asthma by age 6. On the other hand, Boker et al. (15) demonstrated no association between the mode of delivery and asthma incidence. However, this study has some limitations regarding sample size and information about exposition of the neonatal to maternal microflora due to premature rupture of membranes. Therefore, further research should be addressed to answer the questions regarding mode of delivery and asthma incidence.

In addition to mode of delivery, the antibiotics usage during the pregnancy seems alter both the maternal and neonatal microbiomes which may lead to subsequent allergy diseases in childhood (16, 17). Moreover, evidence suggesting that maternal antibiotic usage before and after pregnancy can increase the childhood asthma's risk (18). Furthermore, it should be noted that child exposed to antibiotics in the first days of life can reduced abundance and diversity of *Bifidobacterium* species (18) and increase the abundancy of *Enterobacteriaceae* species (19), which may induce the development of asthma. Therefore, it is important to raise the question about the contributions of antibiotics and infection on microbiome disturbance during and after pregnancy, further studies regarding this topic should be address.

The microbiota colonization of the child may be promoted by maternal gut microbiota *in utero*, after delivery and finally through breastfeeding. Maternal nutrition seems to play an important role on gut microbiota composition alterations in the child. Mother who intake a high-fat diet alters the child microbiome during pregnancy and lactation. The high-fat diet induces increase in *Enterococcus* and decreases *Bacteroides* in the third trimester of pregnancy, aside from decrease *Bacteroides* at delivery (20). Moreover, obese breastfeeding mothers has showed *Bacteroides* decreases in breast milk (21) which can induce a risk of asthma development in the early life (22).

On the other hand, human milk from mother who intake adequate nutrition, induces a general health benefits for the child and the World Health Organization recommends breastfeeding for at least 6 months after birth. Le Doare et al. (23) suggested that human milk can provide nutrition for the microbiome and prevent pathogenic bacterial adhesion. However, in nowadays some mothers has been replaced the breastfeeding and/or supplemented with cow's milk formula. In this sense, several studies have been demonstrated that the food sensitization in the early life may be associated with an increased risk of asthma (24) which can be mediated by an inflammatory immune response driven by Th2 cells. Liang et al. (25) demonstrated that neonatal fed by breast milk had an increase in *Bifidobacterium* and *Lactobacillus* and less viruses in stool samples in compared to those fed with cow's milk, which suggest that breastfeeding can be a potential protection against asthma. A Randomized Clinical Trial with a total of 312 newborns in 6 years follow-up, demonstrated that the cow's formula milk should be avoiding in the first 3 days of life especially in neonatal with higher levels of total Immunoglobulin-E (IgE) that can presents food sensitization in the early life (26). On the “Prevention strategies and immune training” section, we can observer that as earlier we introduce the allergic food the child can development a protective allergic effect.

Apart from food, the pollution and smoking exposure can be a risk for allergic sensitization and enhancement the probability of allergic asthma. Zheng et al. (27) collected fecal samples from 21 children in clean and smog days. Air pollution alters the intestinal microbiome in asthmatic children, increasing *Bacteroidetes* and decreasing *Firmicutes*, these changes can be associated with asthma development. Besides air pollution, tobacco smoke exposure *in utero* and after birth may be associated with a risk of respiratory symptoms in childhood (28).

Children of mothers who smoked during their entire pregnancy present with a higher abundance of *Enterobacteriaceae* (29) at birth and increased abundance of *Bacteroides* and *Staphylococcus* at 6 months of age (30).

Therefore, we can suggest that the gut microbiota presents an important role in asthma development, probably due to the transfer of metabolites and immunomodulatory signals to the lung by gut-lung axis. Although are evidence regarding this connection, the appropriate pathway is not well-elucidated. Previous study demonstrated that gut dysbiosis can increase the allergic lung inflammation through both dendritic cells and T cells (31). Further studies should be address in this field. However, knowing the factor risks which induce a gut dysbiosis and may developed asthma, we can trace potential prevention strategies that can be applied during pregnancy and postnatal period.

## Prevention Strategies and Immune Training

Pinning down strategies for asthma prevention in pregnancy and childhood has attracted great interest lately. The identification of potentially modifiable environmental and host risk factors for asthma development appears to be the cornerstone for the paradigm shift from disease treatment toward primary asthma prevention (32). Childhood asthma risk can be dampened by an appropriate maternal asthma control. The latter includes components such as monthly monitoring of lung function, patient education on inhaler technique, avoidance of environmental triggers (e.g., cigarette smoking, pollen, air pollution), and pharmacological treatment of comorbid conditions (e.g., depression, rhinitis, gastroesophageal reflux) (33).

The capacity of immune training the fetus by maternal environment provides possibilities for prevention of asthma after birth. Management of microbial dysbiosis could be a potential target for this training. Maternal diet and nutritional supplementation can shape immune *in utero* regarding to the airway's response later in life. Evidence suggests that a mother who intake high-fiber diet during pregnancy, leads to changes in the microbiota, enhancing T-regulatory cell numbers and function (34). Moreover, recent randomized clinical trials showed positive results on asthma prevention in offspring derived from adequate levels of vitamin D, antioxidants and fish oil intake during pregnancy (35–38).

In the postnatal period, prevention measures include the control of severe neonatal respiratory infections (e.g., respiratory syncytial virus and human rhinovirus), incentive for breastfeeding and enhancing other microbial exposure through the “farm effect,” as endorsed by the hygiene hypothesis (39, 40). Beyond that, after the LEAP study (41), the old-fashioned avoidance allergenic foods strategy from the diets of infants began to be replaced by the tolerance strategy toward early exposure to allergens. This remarkable study demonstrated that early exposure to allergen can increase the levels of allergic-specific IgG and IgG4 which may induce an absence allergic reaction. Afterward, Pitt et al. (42) demonstrated a reduced risk of allergic sensitization following exposure through breast-feeding. In this sense, as early allergic foods are introduced its possible to training the immune system against allergic sensitization and it can be a strategy for asthma prevention.

Furthermore, supplementation with probiotic and vitamin could be a good strategy for immune training. Administration of probiotic—*Lactobacillus rhamnosus-* on postnatal period, showed a reduced risk of childhood asthma (43) probably because the probiotics can modulate the levels of short chain fatty acid and alters the microbiome composition. On the other side, the role of vitamin D on asthma management relies on its effects on immune cell function (44), corticosteroid responsiveness mediated by pathways involving IL-10 (45), IL-17 (46), oxidative stress (47), and airway remodeling (48). Results from observational studies are still mixed and limited, with more studies showing a beneficial effect for supplementation with vitamin D (49).

Prevention strategies involved in the translation of the environmental exposures elucidated in epidemiological studies mainly focus on asthma protective environmental microbial exposures associated with rural lifestyle activities. This led to some preclinical studies with bacterial lysates (ongoing clinical trial NCT02148796) and metabolites, dietary derivatives and helminthic compounds in order to prevent the disease development (50).

Moreover, The Finnish Allergy prevention program (51) describe practical advices regarding early life exposure: (i) Support breastfeeding, with solid foods from 4–6 months, (ii) do not avoid exposure to environmental allergens (foods, pets), (iii) do not smoke, (iv) probiotic bacteria in fermented food or other preparations and (v) Antibiotics should be taken only if is really necessary. All this simple practical approach can shape the immune system during early life and prevent asthma.

## Conclusions

The changes in microbiome composition due to diseases is called *dysbiosis*. Understanding its roles and the immune responses due to this imbalance in asthma are promising both to comprehend the disease pathophysiology and to elaborate preventive strategies. Dietary interventions are considered safe and promising to boost the immune system and attenuate asthma symptomatology in children. Nevertheless, tackling asthma prevention is challenging because of the existing knowledge gap on the immune pathways that predispose some infants to develop asthma and not others. It seems that the beneficial effects resulting from prevention approaches are due to the combination of them, instead of just one strategy. However, further research is needed on observational studies and clinical trials on the effects of using different combined strategies vs. a sole intervention for asthma prevention.

## Author Contributions

LG, JA, AF, AS, GS, DM, and LO conceived the design and concepts. RP, AC, VO, and IC wrote the manuscript. All authors contributed to the editing and revision of the manuscript and approved the submission.

## Conflict of Interest

The authors declare that the research was conducted in the absence of any commercial or financial relationships that could be construed as a potential conflict of interest.
